# 

**Published:** 1893-04

**Authors:** 


### CONTENTS—APRIL, 1893.—VOL. VIII., NO. 10.

ORIGINAL COMMUNICATIONS—

Cerebral Syphilis. Dr. Matthew M. Smith, Austin . . . . . . . . . . 385
Treatment for Fistula in Ano without a Cutting Operation. Dr. T. J. Bennett, Austin . . . . . . . . . . . . . . . . . . . . . . . . . . 390
Observation on a New Role for Ergotine. Dr. T. C. Osborne, Cleburne . . . . . . . . . . . . . . . . . . . . . . . . . . . . . . 393
Case of Sarcoma of the Ileum, etc. Prof. James E. Thompson, Galveston . . . . . . . . . . . . . . . . . . . . . . . . . . . . . . 395
Cocaine in Urethral Surgery. Prof. Samuel E. Milliken, New York . 398

ABSTRACTS OF CURRENT MEDICAL LITERATURE—

Department of Therapeutics. Dr. David Cerna, Galveston . . . . . 401
Department of Practice of Medicine. Prof. A. J. Smith, Galveston . 404
Obstetrics and Diseases of Women. Prof. W. Keiller, Galveston . . 407
Department of Dermatology. Dr. Isadore Dyer, New Orleans . . . 410

SOCIETY NOTES—

Announcement and Program Texas State Medical Association, etc . 412
Austin District Medical Society . . . . . . . . . . . . . . . . . . . . 416

EDITORIAL—

Legal Murder . . . . . . . . . . . . . . . . . . . . . . . . . . 421
The Free-Booter Again . . . . . . . . . . . . . . . . . . . . . . 422
Texas Students . . . . . . . . . . . . . . . . . . . . . . . . . 423

MEDICAL NEWS AND MISCELLANY . . . . . . . . . . . . . . . . . . . 424

Additional Items . . . . . . . . . . . . . . . . . . . . . . . . 42[illegible]

PUBLISHERS' NOTES . . . . . . . . . . . . . . . . . . . . . . . 428

